# Acute hepatitis C in persons infected with the human immunodeficiency virus (HIV): the 'real-life setting' proves the concept

**DOI:** 10.1186/1758-2652-13-S4-P202

**Published:** 2010-11-08

**Authors:** P Ingiliz, MJ Obermeier, L Weitner, C Cordes, A Moll, B Hintsche, F Schlote, S Köppe, C Mayr, A Baumgarten

**Affiliations:** 1 Medical Center for Infectious Diseases, Berlin, Germany; 2 Medical Center for Infectious Diseases, Hamburg, Germany; 3 Praxis City Ost, Berlin, Germany; 4 Praxiszentrum Kaiserdamm, Berlin, Germany; 5 Praxis Hintsche-Klausen, Berlin, Germany; 6 Praxis Turmstrasse, Berlin, Germany; 7 Praxis Köppe/Kreckel, Berlin, Germany; 8 Aerzteforum Seestrasse, Berlin, Germany

## Purpose of the study

The aim of this retrospective analysis was to review treatment decisions and outcomes in HIV positive patients with acute hepatitis C in a routine clinical setting. Outbreaks of acute HCV infection have been described in several cities recently. The epidemic affects mainly MSM who are coinfected with HIV and is supposedly linked to certain sexual risk practices. Here, we compared our findings with current knowledge and recommendations.

## Methods

HIV-positive patients with the diagnosis of acute HCV infection were included in the retrospective analysis. The patients came from outpatient infectious disease centers in northern German cities. We looked at markers of HIV and HCV infection and compared patients who received treatment and those who did not. Treated patients were followed up to 72 weeks.

## Summary of results

Three hundred nineteen HIV-positive patients with acute hepatitis C between 2001 and 2008 and were included in the analysis. All patients were male, 315 (99%) patients were of Caucasian origin, 296 (93%) declared homosexual contacts as a risk factor for HCV infection, intravenous drug use was declared in 3 (1%) cases. Median age at HCV diagnosis was 40 years (range 20-69 years). Median HCV viral load was 1.2 x 106 IU/mL. The HCV genotype distribution was as follows: 222 patients (70%) had genotype 1, 7 (2%) genotype 2, 26 (8%) genotype 3, 59 (18%) genotype 4. The median time of HIV infection was 5.5 years (range 0 to 22.4 years). Median HIV viral load was 110 copies/mL (range 25 to 10x106 copies/mL). The median CD 4 count was 461 cells/mm^3^ (range 55-1331 cells/mm^3^). Two hundred and forty-six patients (77%) received anti-HCV treatment, and 175 (55%) had completed therapy by the time of the analysis. Median treatment duration was 33 weeks (IQR 24.1-49.9). 93 of the 175 treated patients (53%) reached a sustained virological response (SVR). Treatment duration was significantly higher in the SVR group (40.6 weeks vs 26.6 weeks, p<0.0001). Seventy-three patients (23%) did not receive anti-HCV treatment. In 19 of the untreated patients (26%) the hepatitis C virus was cleared spontaneously.

## Conclusions

Our findings indicate that acute hepatitis C in HIV infected patients affects mainly MSM who acquire HCV sexually. Patients had a short duration of HIV infection and a stable immunological situation. In this real-life setting from urban regions in northern Germany, treatment rates appear to be high and effective.

